# Reconfigurable Cilia-Based Magnetic Millirobots for Cooperative Particle Manipulation Through Programmable Assembly in Microfluidics

**DOI:** 10.3390/mi17070834

**Published:** 2026-07-13

**Authors:** Dineshkumar Loganathan, Chia-Yuan Chen

**Affiliations:** 1 Department of Mechanical Engineering, National Cheng Kung University, Tainan 701, Taiwan; dinemon27@gmail.com; 2 Department of Power Mechanical Engineering, National Tsing Hua University, Hsinchu 300, Taiwan

**Keywords:** reconfigurable millirobots, magnetic cilia, programmable assembly, particle manipulation

## Abstract

Reconfigurable robotic systems have emerged as platforms for particle manipulation owing to their adaptability and capability to alter structural configurations according to task requirements. However, achieving programmable particle capture, transportation, and release through cooperative interactions among untethered robots within microfluidic environments remains challenging. In the present study, reconfigurable cilia-based magnetic millirobots (CMMRs) were developed for cooperative particle manipulation through programmable assembly. The platform consisted of multiple CMMRs that were independently actuated using an electromagnetic coil array and assembled into a cooperative structure possessing a central cavity for particle confinement. Through sequential electromagnetic coil activation and pulse-width modulation-based control, programmable assembly, transportation, and disassembly of the CMMRs were achieved. During assembly, self-organization analysis demonstrated that the constituent CMMRs converged toward this configuration, enabling formation of the cooperative structure needed. Subsequently, particle transportation experiments demonstrated the confinement and transportation of particles along predefined trajectories, with trajectory deviations maintained below 5%. Furthermore, μPIV characterization revealed that the assembled structure generated a directional transport corridor with a flow velocity of 4.5 mm s^−1^, providing a hydrodynamic environment for particle transportation compared with individual CMMRs. The demonstrated capabilities can serve as a foundation for reconfigurable untethered robotic systems capable of microhandling operations in lab-on-a-chip environments.

## 1. Introduction

Particle manipulation within microfluidic environments has attracted considerable attention owing to its importance in applications such as biochemical analysis, targeted cargo transport, microassembly, drug screening, cell handling, and lab-on-a-chip systems [1,2,3,4,5,6,7]. Precise control over the capture, transportation, positioning, and release of particles is often required to achieve reliable microscale operations within confined fluidic environments [8,9]. Consequently, numerous particle-manipulation approaches have been developed, including optical, acoustic, electrical, and magnetic techniques [10,11,12,13]. Among these methods, magnetic actuation has emerged as a particularly attractive strategy because wireless, non-contact, and remotely programmable control can be achieved without direct interaction with the manipulated objects [14,15,16,17]. Furthermore, magnetic fields exhibit excellent penetration through biological and microfluidic materials, thereby enabling flexible operation within enclosed environments [18,19,20]. Despite these advantages, achieving programmable particle capture, transportation, and release in an integrated and controllable manner remains challenging, particularly when complex manipulation tasks are required within confined microfluidic domains [21,22,23].

To address these challenges, magnetically actuated microrobots and millirobots have been extensively investigated as active manipulation platforms. Various robotic architectures, including helical swimmers, soft magnetic robots, artificial cilia systems, microswimmers, and bioinspired robotic structures, have been developed for locomotion, cargo transport, fluid manipulation, and microassembly applications [24,25,26]. Through magnetic-field-mediated control, these robotic systems have demonstrated the ability to transport particles, generate localized flow fields, and perform targeted manipulation tasks within microfluidic environments [18,27,28,29,30,31]. Furthermore, the incorporation of soft materials has enabled improved adaptability and compliance during operation in confined spaces [32,33,34]. Nevertheless, the majority of reported manipulation strategies have relied on individual robotic units, where the achievable manipulation capability is inherently constrained by the physical dimensions, actuation force, and functional characteristics of a single robot [35,36]. Consequently, the realization of versatile particle-handling operations involving capture, transportation, and release remains limited when more complex manipulation requirements are encountered [37,38,39].

More recently, cooperative and reconfigurable robotic systems have emerged as promising approaches for overcoming the limitations associated with individual robotic units [40,41,42]. Through cooperative interactions among multiple robots, enhanced manipulation capability, larger operational workspaces, and improved task adaptability can be achieved [40,43,44,45,46]. In particular, reconfigurable robotic systems enable individual robots to dynamically assemble into larger functional structures and subsequently disassemble according to operational requirements [38,47,48]. Such capabilities provide opportunities for on-demand modification of the manipulation architecture without increasing the complexity of the constituent robots [49,50,51]. Although collective robotic assembly, swarm-based locomotion, and cooperative cargo transportation have been demonstrated in several studies, the programmable assembly and disassembly of multiple untethered robotic units for cooperative particle manipulation within microfluidic environments remain relatively unexplored [52,53,54,55,56,57]. Therefore, the development of untethered reconfigurable robotic systems capable of on-demand assembly, cooperative particle manipulation, and controlled disassembly remains an important research objective [40,58,59,60,61].

Motivated by these considerations, a reconfigurable cilia-based magnetic millirobot (CMMR) platform was developed for cooperative particle manipulation within microfluidic environments. Through programmable electromagnetic actuation, multiple untethered CMMRs were independently controlled and dynamically assembled into a reconfigurable cooperative structure capable of particle capture, transportation, and release. Furthermore, programmable assembly, transportation, and disassembly of the constituent CMMRs were achieved through sequential electromagnetic coil activation and pulse-width modulation-based control. The self-organization behavior of the assembled structure was quantitatively characterized, while cooperative particle transportation performance was experimentally evaluated. In addition, the hydrodynamic mechanisms governing the observed manipulation behavior were investigated through μPIV analysis to establish the relationship between the generated flow fields and particle transportation capability. Through these investigations, the proposed platform was demonstrated as a reconfigurable manipulation strategy that combines cooperative assembly, particle handling, and on-demand structural reconfiguration within a single framework. The demonstrated capabilities are expected to contribute toward the development of untethered reconfigurable robotic systems for micro handling and biomedical applications in future lab-on-a-chip technologies.

## 2. Experimental Section

### 2.1. Electromagnetic Actuation and Control System

**Figure 1:**
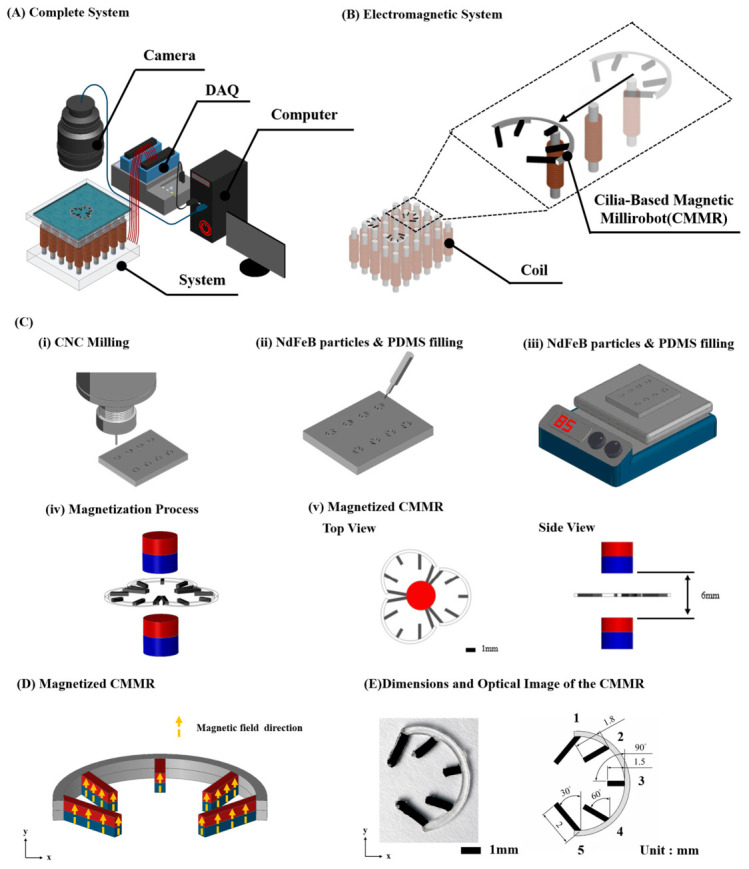
**Design, fabrication, and electromagnetic control of the proposed cilia-based magnetic millirobot (CMMR) platform.** (**A**) Schematic illustration of the complete experimental platform consisting of the electromagnetic actuation system, data acquisition (DAQ) unit, computer-based control interface, vision monitoring system, and microfluidic workspace. (**B**) Electromagnetic actuation principle showing the electromagnetic coil (EC) array and localized magnetic-field generation for programmable CMMR manipulation. (**C**) Fabrication process of the CMMR including (**i**) CNC micromachining of the mold, (**ii**) filling of the NdFeB–PDMS magnetic composite into the ciliary regions, (**iii**) PDMS casting and thermal curing, (**iv**) external magnetization process, and (**v**) fabricated and magnetized CMMR. (**D**) Schematic representation of the magnetized CMMR showing the magnetic moment distribution and magnetic field direction within the ciliary structures. (**E**) Optical image and geometric dimensions of the fabricated CMMR.

To actuate and control the motion of the CMMRs, an in-house electromagnetic coil (EC) array was employed, as illustrated in Figure 1A. Each EC was fabricated by winding 30 turns of copper wire around an iron core measuring 14 mm in length and 1 mm in diameter. A total of 25 ECs were arranged beneath the microfluidic workspace in a rectangular grid configuration to generate localized magnetic fields for programmable robot manipulation. To facilitate efficient magnetic actuation of the CMMRs, the EC array was positioned beneath the microfluidic channel with a vertical separation distance of 1 mm along the z-axis, thereby ensuring effective magnetic coupling between the ECs and the robots. Magnetic actuation was achieved through the coordinated regulation of current supplied to individual ECs. The electromagnetic field was generated and controlled through an integrated system consisting of a data acquisition unit (NI DAQ-9174, National Instruments, Austin, TX, USA), an external direct-current (DC) power supply (GPR-3510HD, Instek, Taipei, Taiwan), and a custom LabVIEW (National Instruments, LabVIEW (version 2019), Austin, TX, USA)-based control interface. Through selective activation of individual ECs and pulse-width modulation (PWM)-based current regulation, localized magnetic field distributions were generated within the workspace. This actuation strategy enabled the independent translational motion, cooperative assembly, transportation, and disassembly of multiple CMMRs. The actuation sequences employed during the assembly, transportation, and disassembly experiments are described in Section 3.2. Meanwhile, the motion of the CMMRs was recorded using a charge-coupled device (CCD) camera (WAT-902H ULTIMATE, WATEC, Tsuruoka City, Japan) equipped with a high-resolution microlens (AF Micro-NIKKOR 60 mm f/2.8D, Nikon, Tokyo, Japan), thereby enabling real-time monitoring and visualization of robot behavior during the experiments.

### 2.2. Material and Fabrication of CMMR

The cilia-based magnetic millirobot (CMMR) was fabricated by employing a composite structure consisting of magnetic and nonmagnetic components. The structural body of the CMMR was fabricated using polydimethylsiloxane (PDMS), whereas the magnetic ciliary structures were fabricated using a magnetic composite composed of neodymium–iron–boron (NdFeB) microparticles embedded within a PDMS matrix [62,63]. PDMS was selected as the base material owing to its biocompatibility, flexibility, optical transparency, elasticity, ease of molding, and widespread applicability in microfluidic systems [62,64,65,66]. The magnetic composite was prepared by blending 5 μm NdFeB particles (MQP-15-7, Magnequench International, Inc., Singapore) with PDMS at a weight ratio of 4:1. The PDMS matrix itself was prepared by mixing the elastomer base and curing agent (Sylgard 184, Dow Corning Corp., Midland, MI, USA) at a weight ratio of 10:1. The fabrication process of the CMMR consisted of six sequential steps, as illustrated in Figure 1C. Initially, a mold containing the desired CMMR geometry was fabricated through a CNC micromachining process. Subsequently, the magnetic composite was selectively introduced into the mold regions corresponding to the ciliary structures, followed by the casting of pure PDMS into the remaining structural regions. Thermal curing was then performed to solidify the composite structure. Following curing, the fabricated CMMR was removed from the mold and subjected to an external magnetization process to establish permanent magnetic moments within the magnetic cilia. The completed CMMR consisted of a C-shaped body integrated with five magnetic cilia of varying lengths positioned along the inner curved boundary of the robot, as shown in Figure 1E. Detailed dimensional information regarding the CMMR geometry and cilia configuration is provided in Figure 1E.

### 2.3. Particle Manipulation Experiments

To evaluate the particle manipulation capability of the proposed reconfigurable CMMR platform, particle capture, transportation, and release experiments were performed within the microfluidic workspace. In contrast to commercially available particles, the target particles employed in the present study were fabricated using polydimethylsiloxane (PDMS) to provide well-defined geometrical characteristics and experimental repeatability. The particle fabrication process was carried out through a molding approach. Initially, particle molds were fabricated using a CNC micromachining process. Subsequently, PDMS (Sylgard 184, Dow Corning Corp., Midland, MI, USA) was prepared by mixing the elastomer base and curing agent at a weight ratio of 10:1 and cast into the fabricated molds. Following thermal curing, the particles were removed from the molds and employed in the manipulation experiments. The fabricated particles possessed a circular geometry with a diameter of 800 μm. Furthermore, the particle manipulation process was recorded using a CCD camera (WAT-902H ULTIMATE, WATEC, Japan) equipped with a high-resolution microlens (AF Micro-NIKKOR 60 mm f/2.8D, Nikon, Japan). The recorded image sequences were subsequently analyzed using ImageJ (version 1.54d) (National Institutes of Health, Bethesda, MD, USA). Particle trajectories and transportation paths were extracted from the recorded images to evaluate the manipulation performance of the reconfigurable CMMR platform. The resulting particle capture, transportation, release behavior, and trajectory characteristics are presented and discussed in Section 3.2 and Section 3.3.

### 2.4. Micro-Particle Image Velocimetry (μPIV) Experiment

To characterize the hydrodynamic behavior generated by both individual and assembled CMMRs, flow-field measurements were performed using micro-particle image velocimetry (μPIV). The objective of this analysis was to quantify the velocity distributions generated by different CMMR configurations and to identify the hydrodynamic mechanisms responsible for particle confinement and transportation. In this study, fluorescent polystyrene tracer particles with a diameter of 8 μm (Microgenics, Inc., Fremont, CA, USA) were uniformly suspended in deionized water and introduced into the microchannel through a precision-controlled syringe pump. After establishing a steady-state condition within the microfluidic environment, the CMMRs were actuated under the prescribed experimental conditions to generate localized fluid motion. The resulting tracer-particle displacements were recorded using a high-speed camera (NR4-S2, IDT, Tallahassee, FL, USA) mounted on a fluorescence microscope (BX60, Olympus Corp., Tokyo, Japan). The acquired image sequences were subsequently processed using Dynamic Studio 2015 (Dantec Dynamics, Skovlunde, Denmark) for quantitative flow-field analysis. Prior to velocity extraction, image preprocessing operations including noise suppression and pixel-intensity normalization were performed to improve image quality. The velocity fields were then calculated using a multipass adaptive cross-correlation algorithm. Specifically, a first-pass interrogation window size of 32 × 32 pixels with 50% overlap was applied for two iterations, followed by a second-pass interrogation window size of 16 × 16 pixels with 50% overlap for an additional two iterations. This hierarchical processing procedure enabled the accurate resolution of localized flow structures generated by the CMMRs. To eliminate spurious vectors, mean-filter-based validation was employed. Velocity vectors exceeding two times the local root-mean-square (RMS) value were identified as outliers and subsequently removed. These vectors were then reinserted if their magnitudes remained within three RMS values of the neighboring vector field. For each experimental condition, 300 image pairs were analyzed under steady-state actuation conditions. The resulting velocity fields were ensemble-averaged to suppress random fluctuations and obtain representative flow-field distributions associated with each CMMR configuration. The ensemble-averaged velocity magnitude and corresponding velocity-vector distributions were subsequently employed to evaluate the hydrodynamic characteristics presented in Section 3.4.

## 3. Results and Discussion

### 3.1. Design, Fabrication, and Electromagnetic Control of the Proposed Cilia-Based Magnetic Millirobots (CMMRs) Platform

A programmable millirobotic platform was developed to investigate cooperative robotic assembly and particle manipulation through cilia-based magnetic millirobots (CMMRs). The primary objective of the developed system was to establish a reconfigurable robotic framework capable of performing particle capture, transportation, and release through the cooperative interaction of multiple robotic units. Unlike conventional gripping systems that rely on permanently connected mechanical structures, the proposed approach was based on the dynamic assembly of multiple untethered CMMRs, thereby enabling the manipulation architecture to be reconfigured according to task requirements. Through this strategy, independent robots were assembled into a larger functional structure for object and particle manipulation and subsequently disassembled following task completion. The overall experimental platform employed in the present study is illustrated in Figure 1A and consisted of a custom-built electromagnetic actuation system, a programmable control interface, a vision monitoring system, and a robotic workspace (a microfluidic channel). Through coordinated current regulation within the electromagnetic coils (ECs), programmable magnetic fields were generated to achieve controlled robot navigation, assembly, and cooperative manipulation. A detailed discussion regarding the electromagnetic actuation strategy and programmable assembly process is provided in Section 3.2. The real-time positions and orientations of the robots were monitored by employing an optical imaging system, thereby enabling the precise control of multi-robot operations within the workspace. Furthermore, the electromagnetic actuation principle employed for robot control is schematically illustrated in Figure 1B. The actuation system consisted of an array of independently addressable ECs that were arranged beneath the microfluidic workspace. Through selective activation of individual coil groups, localized magnetic fields were generated within specific regions of the workspace, thereby enabling spatially selective robot actuation. Consequently, individual CMMRs were independently navigated, positioned, and oriented without requiring physical connections or dedicated onboard power sources. Such a control strategy provided the foundation for programmable assembly and cooperative manipulation operations involving multiple robots. Detailed descriptions regarding the materials and construction of the electromagnetic actuation system are provided in Experimental Section 2.1. Furthermore, the fabrication process employed for CMMR development is schematically presented in Figure 1C. In particular, robot molds were fabricated through a micromachining process, followed by the selective casting of magnetic composite materials and polymeric structural components. Subsequent thermal curing, demolding, and magnetization procedures were performed to obtain the final robot architecture. The structural body of the CMMR was fabricated by employing polydimethylsiloxane (PDMS), whereas the magnetic ciliary structures were fabricated using a NdFeB–PDMS magnetic composite. Detailed fabrication procedures and material compositions are provided in Experimental Section 2.2. The magnetization strategy employed for CMMR actuation is illustrated in Figure 1D. Following fabrication, permanent magnetic moments were established through an external magnetization process, thereby enabling magnetic actuation under the electromagnetic control system. The magnetic moment distribution established within the five magnetic cilia provided spatially distributed magnetic responsiveness throughout the CMMR, thereby enabling coordinated translational and rotational responses under externally generated magnetic fields during cooperative assembly and particle manipulation. The final fabricated CMMR is illustrated in Figure 1E. Each CMMR consisted of a C-shaped body integrated with five magnetic cilia of varying lengths positioned along the inner curved boundary. The C-shaped geometry was selected to facilitate the cooperative assembly of three independently actuated CMMRs into an enclosed reconfigurable structure for particle confinement, transportation, and controlled release. To complement this assembly strategy, cilia lengths were gradually varied (2.0, 1.8, 1.5, 1.8, and 2.0 mm from C1 to C5) to preserve available space within the central manipulation cavity while maintaining magnetic responsiveness in the peripheral regions of the CMMR. In addition, the cilia orientations followed the curvature of the PDMS body, with C1 and C5 positioned at 30°, C2 and C4 at 60°, and C3 at 90° relative to the longitudinal axis of the CMMR, thereby facilitating coordinated orientational adjustment during cooperative assembly. Thus, the developed platform established a programmable framework in which multiple CMMRs were independently controlled, assembled into cooperative structures, and subsequently employed for particle manipulation tasks.

### 3.2. Programmable Assembly, Transportation, and Disassembly of Multiple CMMRs

**Figure 2:**
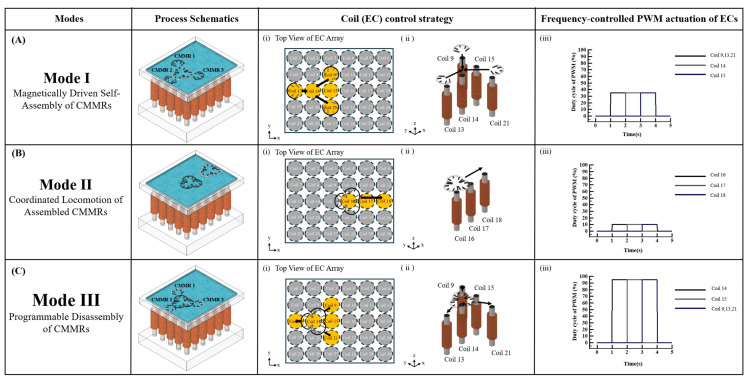
**Programmable assembly, transportation, and disassembly of multiple CMMRs through sequential EC activation.** (**A**) Mode I showing the assembly process in which three independent CMMRs are guided toward a common location to form a reconfigurable cooperative structure. (**i**) Top view of the EC array with the activated coils highlighted in yellow. (**ii**) Activated ECs employed to guide and assemble the CMMRs. (**iii**) Pulse-width modulation (PWM)-based actuation profile corresponding to the assembly sequence. (**B**) Mode II showing transportation of the assembled reconfigurable structure from one location to another within the microfluidic workspace. (**i**) Top view of the EC array with the activated coils highlighted in yellow. (**ii**) Activated ECs employed to translate the assembled structure. (**iii**) PWM-based actuation profile corresponding to the transportation sequence. (**C**) Mode III showing disassembly of the reconfigurable cooperative structure to separate the constituent CMMRs and enable particle release. (**i**) Top view of the EC array with the activated coils highlighted in yellow. (**ii**) Activated ECs employed to separate and reposition the individual CMMRs. (**iii**) PWM-based actuation profile corresponding to the disassembly sequence.

Cooperative robotic systems offer significant advantages for object manipulation because multiple robotic units can collectively perform tasks that are difficult to achieve using individual robots. In particular, reconfigurable assembly enables independent robots to dynamically form larger manipulation structures, thereby increasing operational adaptability while maintaining the simplicity of individual robotic units. Motivated by these advantages, the programmable assembly, transportation, and disassembly of multiple CMMRs were investigated by employing the electromagnetic coil array shown in Figure 2. The overall control strategy was achieved through sequential activation of selected electromagnetic coils (ECs) by employing pulse-width modulation (PWM)-controlled current inputs. By regulating the activation sequence and duty cycle supplied to individual ECs, localized magnetic field distributions were generated to manipulate the position, orientation, and collective behavior of the CMMRs throughout the assembly, transportation, and disassembly processes. Consequently, the same robotic building blocks were transformed from independent units into a reconfigurable cooperative manipulation structure and subsequently returned to their initial independent state following task completion. Specifically, the assembly operation (denoted as Mode I) was performed, as illustrated in Figure 2A and Supplementary Video S1. The objective of this mode was to assemble three independently positioned CMMRs into a reconfigurable cooperative manipulation structure at the location above EC 14. The complete EC array and corresponding coil numbering scheme employed throughout the experiments are presented in Figure 2A(i), whereas the selectively activated ECs utilized for the assembly task are highlighted in Figure 2A(ii). Initially, CMMR 1, CMMR 2, and CMMR 3 were positioned above EC 9, EC 13, and EC 21, respectively. At the initial time step, these ECs were activated by employing a PWM duty cycle of 35%, as shown in Figure 2A(iii), thereby maintaining the robots at their respective starting locations. Subsequently, EC 15, positioned between the initial robot locations and the target assembly region, was activated. The localized magnetic field generated by EC 15 attracted the neighboring CMMRs toward the central region, while the distributed magnetic moments within the five magnetic cilia simultaneously generated coordinated translational and rotational responses, thereby progressively reducing the separation distance between the robots and facilitating cooperative assembly. This intermediate assembly stage is reflected by the corresponding PWM modulation profile shown in Figure 2A(iii). Finally, EC 14 was activated during the final stage of the sequence, resulting in the convergence of all three CMMRs toward the target location to establish an enclosed reconfigurable cooperative structure capable of subsequent particle confinement, transportation, and controlled release. As the robots assembled above EC 14, a stable reconfigurable cooperative structure possessing a central cavity was formed. This reconfigurable assembled structure subsequently served as the manipulation unit employed for particle grasping operations. Following assembly, transportation of the reconfigurable assembled structure (denoted as Mode II) was performed, as illustrated in Figure 2B and Supplementary Video S1. The objective of this mode was to translate the assembled CMMRs from their initial position above EC 16 to a new target location above EC 18 while preserving structural integrity. The EC array and selectively activated ECs employed during transportation are shown in Figure 2B(i,ii), respectively. Initially, EC 16 was activated with a PWM duty cycle of 10%, thereby maintaining the assembled structure at its starting location. Subsequently, EC 17 was activated to generate an intermediate magnetic attraction force, resulting in displacement of the assembled structure toward the target region. Finally, EC 18 was activated during the last stage of the sequence, thereby attracting the reconfigurable cooperative structure toward its final destination. The sequential transfer of magnetic actuation from EC 16 to EC 17 and subsequently to EC 18 can be clearly observed from the PWM modulation profile shown in Figure 2B(iii). Through this controlled actuation strategy, the assembled CMMRs were translated as a single cooperative manipulation unit while preserving the enclosed cooperative structure established during assembly throughout particle transportation. After transportation was completed, controlled disassembly of the reconfigurable cooperative structure (denoted as Mode III) was performed, as illustrated in Figure 2C and Supplementary Video S1. The objective of this mode was to separate the assembled CMMRs and return them to their original locations, thereby enabling the release of the potential particle confined within the assembled structure. The corresponding EC array and selectively activated ECs are shown in Figure 2C(i,ii), respectively. Initially, the assembled structure was positioned above EC 14. At the first time step, EC 14 was activated using a PWM duty cycle of 95%, thereby maintaining the assembled configuration at the designated release location. Subsequently, EC 15 was activated with a PWM duty cycle of 95%, initiating separation of the outer CMMRs from the central assembly. Finally, EC 9, EC 13, and EC 21 were sequentially activated to attract CMMR 1, CMMR 2, and CMMR 3 back toward their respective initial locations. The corresponding PWM modulation profile shown in Figure 2C(iii) illustrates the redistribution of actuation power among the selected ECs during the disassembly process. As the robots progressively moved away from the central assembly location, the enclosed cavity formed during Mode I was opened, thereby enabling release of the confined particle. The assembly, transportation, and disassembly experiments described above were performed by employing a supplied current of 2 A together with a 1 s PWM switching interval between successive EC activations. To evaluate the dynamic response of the proposed CMMR platform under these operating conditions, additional experiments were performed to quantify the translational velocity and the response delay between EC activation and the onset of CMMR motion. The measured response delay remained significantly shorter than the selected PWM switching interval, thereby enabling the CMMRs to respond to each activated EC before the subsequent switching event. The corresponding dynamic response characterization is presented in Supplementary Section S1 and Figure S1. Overall, the three operational modes demonstrate a programmable electromagnetic control strategy capable of achieving the assembly, transportation, and disassembly of multiple CMMRs through sequential EC activation and PWM-based actuation. Through appropriate regulation of the activated ECs and corresponding duty-cycle modulation profiles, independent CMMRs were transformed into a stable reconfigurable cooperative structure, translated as a single manipulation unit, and subsequently disassembled in a controlled manner to enable particle release. Furthermore, this demonstrated strategy illustrates how programmable electromagnetic actuation and cooperative structural reconfiguration collectively governed the particle manipulation capability of the proposed platform while maintaining independent control of each CMMR. In the present study, a three-CMMR cooperative configuration was employed to manipulate particles within the investigated size range. Nevertheless, the proposed assembly strategy is inherently scalable, as the number of cooperatively assembled CMMRs can be adjusted based on the dimensions of the target object. Such scalability requires an expansion of the electromagnetic coil array to provide additional, independently addressable actuation regions for robot navigation, cooperative assembly, and coordinated manipulation. Consequently, larger cooperative assemblies can be achieved by expanding the external electromagnetic actuation system while preserving the programmable assembly strategy presented in this work. Such reconfigurable assembly behavior establishes the operational foundation for cooperative particle capture, transportation, and release, which are experimentally demonstrated in the following section.

### 3.3. Self-Organization and Cooperative Particle Transportation of Reconfigurable CMMRs

**Figure 3:**
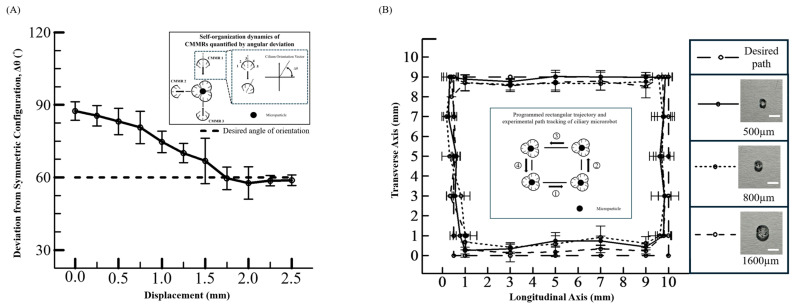
**Self-organization and cooperative particle transportation of the reconfigurable CMMRs.** (**A**) Quantitative evaluation of the self-organization process based on the angular deviation (Δθ) of CMMR 1 during cooperative assembly. The angular deviation was determined from the orientation of the third cilium with respect to the longitudinal axis of the workspace. The dashed line represents the required assembly orientation (60°) for forming the enclosed reconfigured structure under the selected initial robot arrangement. The inset illustrates the assembled cooperative configuration and the definition of the angular deviation. Error bars represent the standard deviation obtained from five independent experiments. (**B**) Cooperative transportation performance of the assembled CMMRs for circular PDMS particles with diameters of 500 μm, 800 μm, and 1600 μm. The assembled cooperative structure transported the enclosed particles along a predefined rectangular trajectory consisting of four sequential paths (Path 1–Path 4), as illustrated in the inset. The inset on the right shows the optical images of the circular PDMS particles used in the transportation experiments. Error bars represent the standard deviation obtained from five independent transportation experiments for each particle size, demonstrating the repeatability of the proposed cooperative transportation strategy.

The cooperative manipulation capability of the proposed CMMRs was evaluated through self-organization, particle transportation, and controlled particle release experiments, as presented in Figure 3 and Supplementary Video S1. During the self-organization process, three independently controlled CMMRs (CMMRs 1 to 3) were assembled into an enclosed reconfigured structure capable of cooperative particle manipulation. Figure 3A illustrates the angular evolution of the CMMRs during the assembly process. Initially, CMMR 2 was maintained at an orientation of 0°, whereas CMMR 1 and CMMR 3 were translated toward the target assembly location while simultaneously undergoing rotational reorientation. Under the selected initial arrangement of the three CMMRs, an orientation of 60° was required for both CMMR 1 and CMMR 3 to form the enclosed reconfigured structure shown in the inset of Figure 3A. This orientation was achieved through rotational and translational reorientation from their initial orientation of 90°. Since the required orientation was governed by the initial spatial arrangement of the CMMRs, different initial robot configurations would require different orientation angles to establish the corresponding enclosed structure. This assembly behavior was quantified by measuring the angular deviation of CMMR 1 with increasing translational displacement, where the orientation was determined from the third cilium with respect to the longitudinal axis of the workspace. Since CMMR 3 was positioned symmetrically opposite CMMR 1, an identical rotational behavior was observed in the opposite quadrant during the assembly process. As shown in Figure 3A, the angular deviation gradually converged toward the required assembly orientation as the CMMRs approached the target assembly location. The self-organization experiment was repeated five times to evaluate the repeatability of the proposed control strategy. An average standard deviation of 4.89° was obtained for the measured angular deviation at the final assembly position, demonstrating good repeatability of the cooperative assembly process. Meanwhile, the translational and rotational motions of the CMMRs were governed by the magnetic force and magnetic torque generated under the externally applied magnetic field produced by the electromagnetic coil array. The theoretical derivation of the governing magnetic force and torque equations is presented in Supplementary Materials S2. Based on this theoretical framework, numerical simulations were performed to predict the magnetic field distribution generated by the electromagnetic coil array, followed by experimental validation through Hall-effect sensor measurements. The corresponding numerical prediction and experimental validation are presented in the Supplementary Materials S3 and Figure S2. Following self-organization, the assembled CMMRs were subsequently employed as a cooperative manipulation structure for particle transportation (Figure 3B and Supplementary Video S1). The transportation behavior was evaluated by guiding the enclosed particle along a predefined rectangular trajectory composed of four sequential paths, denoted as Path 1–Path 4, as illustrated in the inset of Figure 3B. Specifically, the assembled structure was translated horizontally along Path 1, vertically along Path 2, horizontally in the opposite direction along Path 3, and finally vertically along Path 4 to complete one transportation cycle. The optical images of the circular PDMS particles employed for the transportation experiments, with diameters of 500 μm, 800 μm, and 1600 μm, are shown in the right inset of Figure 3B. The transportation capability was first evaluated for these three particle sizes. For all three particle sizes, the assembled CMMRs successfully enclosed the particle throughout the transportation process while closely following the prescribed rectangular trajectory. The transportation experiments were repeated five times for each particle size, and the corresponding trajectory deviations are presented in Figure 3B. The average trajectory deviations for the 500 μm particle were 0.58 ± 0.17 mm, 9.77 ± 0.27 mm, 9.13 ± 0.21 mm, and 0.59 ± 0.27 mm along Path 1–Path 4, respectively. Similarly, the average trajectory deviations for the 800 μm particle were 0.70 ± 0.35 mm, 9.69 ± 0.55 mm, 9.3 ± 0.27 mm, and 0.61 ± 0.28 mm, whereas those for the 1600 μm particle were 0.36 ± 0.23 mm, 9.77 ± 0.33 mm, 8.93 ± 0.37 mm, and 0.65 ± 0.18 mm, respectively. These observed trajectory deviations were attributed to the combined influence of localized hydrodynamic disturbances generated during repeated translation of the assembled cooperative structure, together with the measured response delay between electromagnetic coil activation and the onset of CMMR motion (Supplementary Figure S1), which introduced minor transient positional offsets during successive electromagnetic coil switching. Since the present platform employed an open-loop electromagnetic actuation strategy, these transient positional offsets were not actively corrected during transportation and therefore contributed to the experimentally observed trajectory deviations. Previous studies have demonstrated that closed-loop electromagnetic control strategies incorporating real-time visual feedback and adaptive navigation can further improve positioning accuracy by compensating for such transient positioning errors during robotic manipulation [67,68,69]. Meanwhile, the measured trajectory deviations across the four transportation paths demonstrated the repeatability of the proposed cooperative transportation strategy for different particle sizes. In addition to circular particles, the versatility of the proposed CMMR platform was further demonstrated through the manipulation of a 1600 μm square PDMS particle, and the corresponding experimental results are presented in Supplementary Figure S3. Successful transportation of both circular and square particles demonstrated that the proposed cooperative enclosure was not restricted to a single particle geometry. The particle manipulation capability of the proposed CMMR platform was primarily governed by the geometric relationship between the enclosed manipulation space formed by the assembled CMMRs and the size of the manipulated particle. As the particle dimensions increased relative to the available enclosure, the ability of the assembled CMMRs to completely enclose the particle gradually decreased, limiting the cooperative manipulation performance. Consequently, the maximum manipulable particle size was determined by the enclosure geometry established by the assembled CMMRs rather than by the electromagnetic control strategy itself. Meanwhile, though the transportation experiments demonstrated the manipulation capability of the reconfigurable cooperative structure, the underlying physical mechanisms governing particle confinement and transportation remain closely associated with the hydrodynamic interactions generated by the assembled CMMRs. Therefore, to obtain further insight into the flow characteristics responsible for the observed manipulation behavior, detailed flow-field characterization studies were subsequently performed and are discussed in the following section.

### 3.4. Hydrodynamic Mechanisms Governing Cooperative Particle Transportation

**Figure 4:**
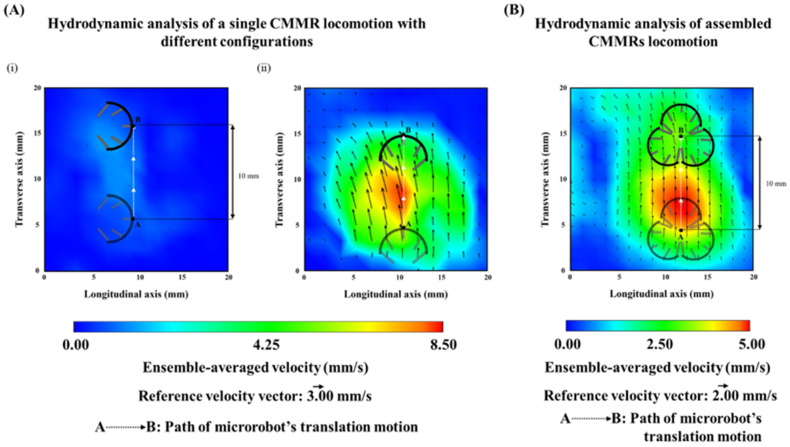
**μPIV characterization of the flow-field dynamics generated by individual and assembled CMMRs.** (**A**) Ensemble-averaged velocity contours and velocity-vector distributions generated by an individual CMMR along the transportation direction extending from Point A to Point B. (**i**) Individual CMMR with the orientation vector of Cilium 3 aligned with the positive x-axis (0° orientation), generating an average flow velocity of 1.1 mm s^−1^. The velocity field was ensemble-averaged over the complete transportation process, which required an average duration of 10.6 s. (**ii**) Individual CMMR with the orientation vector of Cilium 3 oriented at 90°, generating an average flow velocity of 8.5 mm s^−1^. The velocity field was ensemble-averaged over the complete transportation process, which required an average duration of 2.4 s. (**B**) Ensemble-averaged velocity contour and velocity-vector distribution generated by the assembled reconfigurable CMMR structure. A confined and directional transport corridor is established between Points A and B with an average flow velocity of 4.5 mm s^−1^. The velocity field was ensemble-averaged over the complete transportation process, which required an average duration of 4.0 s.

Hydrodynamic interactions play a critical role in robotic particle manipulation because the generated flow fields directly influence particle confinement, transportation, and release processes. In particular, the spatial distribution and magnitude of the induced flow field determine whether manipulated particles remain confined within the intended manipulation region or are dispersed into the surrounding fluid domain. Therefore, characterization of the flow-field dynamics generated by the CMMRs is essential for understanding the physical mechanisms responsible for the cooperative particle transportation behavior demonstrated in the preceding sections. To investigate these hydrodynamic characteristics, μPIV measurements were performed for both individual and assembled CMMRs, as illustrated in Figure 4. Detailed descriptions regarding the μPIV setup, tracer particles, image acquisition procedures, and velocity-field reconstruction methods are provided in the Experimental section (Section 2). For consistency with the particle transportation experiments presented in Section 3.3, the flow-field analysis was performed along the transportation direction extending from Point A to Point B, as shown in Figure 4. During each experiment, the CMMRs were actuated until the target location (Point B) was reached, and the corresponding μPIV image sequences acquired throughout the transportation process were employed for flow-field reconstruction. Subsequently, the velocity fields were ensemble-averaged over the complete transportation duration for each configuration and used to evaluate the generated hydrodynamic environment. Initially, the flow-field characteristics generated by an individual CMMR were investigated under two different orientations, as shown in Figure 4A(i,ii). In the first case (Figure 4A(i)), the orientation of the central cilium (Cilium 3) was aligned with the positive x-axis, corresponding to an orientation angle of 0°. Under this condition, only weak flow disturbances were generated within the surrounding fluid domain. As shown in Figure 4A(i), the ensemble-averaged velocity remained low, reaching a maximum value of 1.1 mm s^−1^. Furthermore, the velocity vectors revealed the absence of a clearly defined transport pathway between Points A and B. Consequently, the generated hydrodynamic force would be insufficient to effectively transport and maintain the confinement of particles during manipulation operations. Subsequently, a second individual-CMMR configuration was investigated by rotating the robot so that the orientation of Cilium 3 was 90°, as shown in Figure 4A(ii). Under this condition, substantially stronger flow disturbances were generated throughout the microfluidic channel. The corresponding ensemble-averaged velocity increased significantly and reached a maximum value of 8.5 mm s^−1^. Furthermore, the velocity vectors indicate that the generated flow occupied a considerably larger region compared with the previous case, extending across a substantial portion of the fluid domain. Although such a flow field provided stronger fluid propulsion, the generated hydrodynamic environment lacked spatial confinement and directional control. Consequently, particles subjected to this flow field would experience widespread fluid disturbances and could be displaced away from the intended transportation path. Therefore, despite the higher flow velocity, the generated flow field would not be favorable for controlled particle manipulation, transportation, and targeted release applications. To address these limitations, the flow-field characteristics generated by the assembled reconfigurable cooperative structure were subsequently investigated, as shown in Figure 4B. Unlike the individual CMMR configurations, the assembled structure generated a highly localized and directional flow pathway extending between Points A and B. The corresponding velocity vectors revealed a confined transport corridor concentrated within the central region of the assembled structure. Furthermore, the ensemble-averaged velocity reached a maximum value of 4.5 mm s^−1^, representing an intermediate flow magnitude between the two individual-CMMR cases. Importantly, the generated flow field simultaneously provided sufficient hydrodynamic strength for particle transportation while avoiding the excessive flow spreading observed in the 90° individual-CMMR configuration. As a result, a narrow and directed transport corridor was established between Points A and B, thereby promoting particle confinement within the central cavity of the reconfigurable cooperative structure during transportation. To further correlate the μPIV analysis with experimentally observed particle transport behavior, additional particle-tracking experiments were performed to track the confined particle along the same transport path investigated in the μPIV measurements. The corresponding particle trajectories obtained from five independent experiments are presented in Supplementary Figure S4. An average trajectory deviation of 0.23 ± 0.07 mm was measured from the desired transportation path, demonstrating that the confined particle remained within the transportation corridor generated by the assembled CMMRs throughout the transportation process. These experimental observations were consistent with the μPIV results, confirming that the localized flow field generated by the assembled structure continuously guided the particle along the central transportation corridor while minimizing lateral displacement. Such behavior directly explains the successful particle manipulation results presented in Section 3.3, where particles remained confined within the assembled structure while being transported along predefined trajectories. Meanwhile, this direct comparison among the three investigated configurations further highlights the hydrodynamic advantages of the reconfigurable cooperative structure. Relative to the 0° individual-CMMR configuration, the assembled structure generated a 4.5-fold increase in maximum ensemble-averaged velocity, thereby providing sufficient hydrodynamic strength for particle transportation. Meanwhile, compared with the 90° individual-CMMR configuration, the assembled structure reduced the maximum velocity by 41%, thereby mitigating excessive flow spreading throughout the microfluidic domain. Consequently, the assembled structure established a balanced hydrodynamic environment in which adequate transport forces were generated while maintaining spatial confinement of the induced flow field. Such a combination of moderate flow magnitude and localized transport pathways is particularly advantageous for particle manipulation applications because it promotes controlled transportation while reducing unintended particle dispersion into surrounding regions. The observed hydrodynamic characteristics indicate that cooperative assembly of multiple CMMRs not only modifies the geometric configuration of the manipulation platform, but also fundamentally alters the surrounding flow-field distribution. Through hydrodynamic coupling among the constituent CMMRs, a confined and directional transport corridor was generated that could not be achieved using individual CMMRs alone. Consequently, the assembled reconfigurable cooperative structure provided a more favorable hydrodynamic environment for particle capture, confinement, transportation, and subsequent release.

## 4. Conclusions

In this work, a reconfigurable particle manipulation platform based on cilia-based magnetic millirobots (CMMRs) was developed and experimentally demonstrated. Through programmable electromagnetic actuation, independent CMMRs were assembled into a cooperative manipulation structure capable of particle capture, transportation, and release. By employing sequential electromagnetic coil activation and PWM-based regulation, the reversible assembly, transportation, and disassembly of multiple CMMRs were successfully achieved. Furthermore, self-organization analysis demonstrated that the constituent CMMRs progressively converged toward the desired assembly configuration during cooperative structure formation. As a result, the assembled structure functioned as a unified manipulation platform, enabling confined particles to be transported along predefined trajectories. To further understand the mechanisms governing this manipulation behavior, μPIV analysis was performed, which revealed that the assembled reconfigurable structure generated a confined and directional transport corridor with a balanced flow magnitude. Consequently, a favorable hydrodynamic environment for particle confinement and transportation was established compared with individual CMMRs. The proposed platform demonstrated the potential of reconfigurable cooperative robotic systems for programmable particle manipulation and provided a foundation for future applications involving cargo transport, particle handling, and microassembly operations within confined fluidic environments.

## 5. Limitations

The proposed CMMR platform was developed to achieve programmable self-organization, cooperative particle transportation, and controlled particle release through the cooperative assembly of three independently actuated CMMRs under the investigated experimental conditions. The reported results were obtained by employing the three-CMMR cooperative configuration operating in a single working fluid (water). Successful cooperative particle manipulation was demonstrated for circular PDMS particles with diameters of 500 μm, 800 μm, and 1600 μm, as well as a 1600 μm square PDMS particle. However, reliable manipulation of 2000 μm circular (Supplementary Figure S5) and square particles was not achieved because the available enclosure formed by the assembled CMMRs was insufficient to completely enclose particles of this size. These observations indicate that the particle manipulation capability of the proposed platform was primarily governed by the geometric relationship between the cooperative enclosure formed by the assembled CMMRs and the dimensions of the manipulated particle. Consequently, the maximum manipulable particle size was determined by the enclosure geometry established by the three-CMMR cooperative configuration. In addition, although the programmable assembly sequence remained governed by the sequential activation of the electromagnetic coil array, the assembly and transportation dynamics of the CMMRs were expected to depend on the balance between the magnetic driving force and the hydrodynamic resistance exerted by the surrounding fluid. Consequently, an increase in fluid viscosity is expected to increase the hydrodynamic resistance acting on the CMMRs, thereby reducing their translational velocity and increasing the assembly and particle transportation times under identical magnetic actuation conditions. Conversely, lower fluid viscosities are expected to reduce the hydrodynamic resistance and facilitate faster translational motion. Since the influence of fluid viscosity was not experimentally investigated in the present study, the reported assembly and transportation performances should be interpreted within the investigated fluid conditions.

## Data Availability

The data that support the findings of this study are available from the corresponding author upon reasonable request.

## Supplementary Materials

The following supporting information can be downloaded at: https://www.mdpi.com/article/10.3390/mi17070834/s1, Text S1: Dynamic Response Characterization of the Proposed CMMR Platform; Text S2: Theoretical Analysis of Magnetic Force and Torque Governing the Self-Organization of CMMRs; Text S3: Numerical Prediction and Experimental Validation of the Magnetic Field; Text S4: Particle-Tracking Analysis of Cooperative Particle Transportation; Figure S1: Dynamic response characterization of the proposed CMMR platform under different supplied currents; Figure S2: Numerical prediction and experimental validation of the magnetic field generated by the electromagnetic coil employed for cooperative self-organization of the CMMRs; Figure S3: Cooperative transportation of a 1600 μm square PDMS particle employing the assembled CMMRs; Figure S4: Particle-tracking analysis of cooperative particle transportation performed using the assembled CMMRs; Figure S5: Sequential optical images illustrating the attempted cooperative manipulation of a 2000 μm circular PDMS particle using the assembled CMMRs; Table S1: Comparison of representative magnetic collective robotic systems reported in the literature with the proposed CMMR platform based on reconfigurability, transport velocity, manipulated object characteristics, manipulation mode, manipulation capacity, and cooperative cargo enclosure capability. References [70,71,72,73,74,75] are cited in the supplementary materials; Video S1: Programmable assembly, transportation, and disassembly of multiple CMMRs for particle manipulation task.

## References

[1] Gimondi S., Ferreira H., Reis R.L., Neves N.M. (2023). Microfluidic Devices: A Tool for Nanoparticle Synthesis and Performance Evaluation. ACS Nano.

[2] Yang K.-H., Loganathan D., Chen M.-L., Sahadevan V., Chen C.-Y., Chen C.-Y. (2024). Enhancement of zebrafish sperm activation through microfluidic mixing induced by aquatic microrobots. Microfluid. Nanofluid..

[3] Ma L., Zhao X., Hou J., Xiao Y., Lu X., Chen Z., Wei J., Hao N. (2026). Droplet microfluidics for biomedical applications: Emerging trends and future developments. Microsyst. Nanoeng..

[4] Sahadevan V., Loganathan D., Chuang Y., Feng Lo C., Chen C.Y., Chen C.Y. (2023). Synergetic benefits of microfluidics using artificial cilia and ZnO/SnFe2O4 for the degradation of pollutants. Mater. Chem. Phys..

[5] Kanniyappan H., Paramasivan M., Narayanaswamy V., Konety R., Perumal G., Lin Y., Badhe R.V., Mathew M.T. (2025). Microfluidics in biomedical research and its application in orthopedics: A perspective review. Microfluid. Nanofluid..

[6] Oushyani Roudsari Z., Esmaeili Z., Nasirzadeh N., Heidari Keshel S., Sefat F., Bakhtyari H., Nadri S. (2025). Microfluidics as a promising technology for personalized medicine. Bioimpacts.

[7] Sharma P.K., Loganathan D., Chen M.-L., Lu Y.-H., Wang P.-H., Chen C.-Y. (2025). Cognitive dynamics of drug-mediated zebrafish under sound stimuli in a microfluidic environment. Biomicrofluidics.

[8] Das A., Prajapati P. (2025). Navigating pharmaceuticals: Microfluidic devices in analytical and formulation sciences. Discov. Chem..

[9] Zhang J., Zhang X., Zhang Y., Yang X., Guo L., Man C., Jiang Y., Zhang W., Zhang X. (2025). Emerging biosensors integrated with microfluidic devices: A promising analytical tool for on-site detection of mycotoxins. npj Sci. Food.

[10] Zhang S., Wang Y., Onck P., den Toonder J. (2020). A concise review of microfluidic particle manipulation methods. Microfluid. Nanofluid..

[11] Tenje M., Fornell A., Ohlin M., Nilsson J. (2018). Particle Manipulation Methods in Droplet Microfluidics. Anal. Chem..

[12] Yiannacou K., Sariola V. (2023). Acoustic Manipulation of Particles in Microfluidic Chips with an Adaptive Controller that Models Acoustic Fields. Adv. Intell. Syst..

[13] Sriphutkiat Y., Zhou Y. (2017). Particle manipulation using standing acoustic waves in the microchannel at dual-frequency excitation: Effect of power ratio. Sens. Actuators A Phys..

[14] Yang Z., Zhang L. (2020). Magnetic Actuation Systems for Miniature Robots: A Review. Adv. Intell. Syst..

[15] Shen H., Cai S., Wang Z., Ge Z., Yang W. (2023). Magnetically driven microrobots: Recent progress and future development. Mater. Des..

[16] Son C., Yang Z., Kim S., Ferreira P.M., Feng J., Kim S. (2023). Bidirectional Droplet Manipulation on Magnetically Actuated Superhydrophobic Ratchet Surfaces. ACS Nano.

[17] Chen C.-Y., Chien T.-C.C., Mani K., Tsai H.-Y. (2016). Axial orientation control of zebrafish larvae using artificial cilia. Microfluid. Nanofluid..

[18] Gijs M.A.M., Lacharme F., Lehmann U. (2010). Microfluidic Applications of Magnetic Particles for Biological Analysis and Catalysis. Chem. Rev..

[19] Ju X., Velluvakandy R., Wu X., Merlos Rodrigo M.A., Heger Z., Bendíčková K., Frič J., Pumera M. (2026). Liquid Metal Microrobots for Magnetically Guided Transvascular Navigation. Adv. Mater..

[20] Kutluk H., Viefhues M., Constantinou I. (2024). Integrated Microfluidics for Single-Cell Separation and On-Chip Analysis: Novel Applications and Recent Advances. Small Sci..

[21] Gong L., Cretella A., Lin Y. (2023). Microfluidic systems for particle capture and release: A review. Biosens. Bioelectron..

[22] Dang Y., Hu S., Ou Z., Zhang Q. (2023). Microparticle Manipulation Performed on a Swirl-Based Microfluidic Chip Featured by Dual-Stagnation Points. Langmuir.

[23] Sebastián V. (2026). Microfluidic reactors for the synthesis of inorganic and hybrid nanoparticles for drug delivery. Adv. Drug Deliv. Rev..

[24] Pramanik R., Verstappen R.W.C.P., Onck P.R. (2024). Nature-inspired miniaturized magnetic soft robotic swimmers. Appl. Phys. Rev..

[25] Zhou H., Mayorga-Martinez C.C., Pané S., Zhang L., Pumera M. (2021). Magnetically Driven Micro and Nanorobots. Chem. Rev..

[26] Kim Y., Zhao X. (2022). Magnetic Soft Materials and Robots. Chem. Rev..

[27] Chakrabarty D., Chakraborty N., Ganguly R. (2026). Manipulating magnetic fluid droplets on a configurable, multitasking surface microfluidic application. J. Magn. Magn. Mater..

[28] Song J., Guo Y. (2026). Magnetic Field Driven Microrobot Based on Hydrogels. Adv. Robot. Res..

[29] Subendran S., Wang C.-F., Loganathan D., Lu Y.-H., Chen C.-Y. (2022). An aquatic microrobot for microscale flow manipulation. Sci. Rep..

[30] Panigrahi B., Chen C.-Y. (2019). Microfluidic retention of progressively motile zebrafish sperms. Lab Chip.

[31] Loganathan D., Wang P.-H., Lu Y.-H., Chen C.-Y. (2026). Programmable microfluidics for zebrafish larvae manipulation using multiple magnetic microrobots. Sens. Actuators Rep..

[32] Liu Q., Peng Z., Sun C., Yang H. (2026). Recent advances in multifunctional soft robots: A materials–structures–systems co-design perspective for synergistic integration. FlexMat.

[33] Omiyale B.O., Akinsola O.F., Ashraf M.A., Olaiya N.G., Ogbeyemi A., Zhang W.C. (2026). The rapid rise of soft robotics in surgical operations: Trends, challenges, and future directions. Robot. Auton. Syst..

[34] Su J., He K., Li Y., Tu J., Chen X. (2025). Soft Materials and Devices Enabling Sensorimotor Functions in Soft Robots. Chem. Rev..

[35] Nahavandi S., Alizadehsani R., Nahavandi D., Lim C.P., Kelly K., Bello F. (2024). Machine learning meets advanced robotic manipulation. Inf. Fusion.

[36] Nazari K., Mandil W., Santello M., Park S., Ghalamzan-E A. (2025). Bioinspired trajectory modulation for effective slip control in robot manipulation. Nat. Mach. Intell..

[37] Zhang S., Zhang R., Wang Y., Onck P.R., den Toonder J.M.J. (2020). Controlled Multidirectional Particle Transportation by Magnetic Artificial Cilia. ACS Nano.

[38] Loganathan D., He D.-M., Chen K.-W., Chen C.-Y. (2026). Untethered Magnetic Microswimmers for Targeted Particle Transport and Flow Manipulation. Adv. Mater. Technol..

[39] Feng M., Shen X., Bian X. (2026). A covered liquid bridge structure for the manipulation and self-assembly of small objects. Colloids Surf. A Physicochem. Eng. Asp..

[40] Li Z., Qi Z., Wu Z., Zhang L., Xu Q. (2026). Cooperative integrated surgical robots for high-performance targeted therapies. Innovation.

[41] Si B., Chang L., Li S., Ding Z., Xie G. (2026). Self-reconfigurable robotic fish swarms: Collective achievement of diverse locomotion and challenging aquatic tasks. Sci. Adv..

[42] Raj R., Song Q.-C., Juang J.-Y. (2026). Multi-Material Additive Manufacturing of Soft Robotic Systems: A Comprehensive Review. Adv. Robot. Res..

[43] Morandini S., Currò F., Parlangeli O., Pietrantoni L. (2025). Collaborative Robots Adapting Their Behavior Based on Workers’ Psychological States: A Systematic Scoping Review. Hum. Behav. Emerg. Technol..

[44] Osa N., Lasa G., Mazmela M., Apraiz A., Escallada O. (2026). Emerging Trends in Cognitive Abilities and Their Impact on Human-Robot Collaboration: A Systematic Literature Review. Int. J. Soc. Robot..

[45] Ketelbuters L., Engelen B., Dekker I., Kellens K. (2026). From Cooperative Dual-Arm Manipulators to Cooperative Multi-Arm Manipulators—Where Are We Standing Today?. Robotics.

[46] Sahadevan V., Panigrahi B., Chen C.Y. (2022). Microfluidic Applications of Artificial Cilia: Recent Progress, Demonstration, and Future Perspectives. Micromachines.

[47] Pasumarthi R., Samarakoon S.M.B.P., Elara M.R., Sheu B.J. (2025). Determining optimum assembly zone for modular reconfigurable robots using multi-objective genetic algorithm. Sci. Rep..

[48] Asif M.E., Rastegarpanah A., Stolkin R. (2024). Robotic disassembly for end-of-life products focusing on task and motion planning: A comprehensive survey. J. Manuf. Syst..

[49] Li Y., Jiang P., Chai C., Zhang X., Liu C. (2025). A framework for robotic manipulation tasks based on multiple zero shot models. Sci. Rep..

[50] Hedayati H., Suzuki R., Rees W., Leithinger D., Szafir D. (2022). Designing Expandable-Structure Robots for Human-Robot Interaction. Front. Robot AI.

[51] Dhanda M., Rogers B.A., Hall S., Dekoninck E., Dhokia V. (2025). Reviewing human-robot collaboration in manufacturing: Opportunities and challenges in the context of industry 5.0. Robot. Comput.-Integr. Manuf..

[52] Yin H., Li Y., Wu G., Guo Y. (2026). The scale matters: A review on stimuli-responsive microrobots categorized by scale for biomedical applications. Responsive Mater..

[53] Koleoso M., Feng X., Xue Y., Li Q., Munshi T., Chen X. (2020). Micro/nanoscale magnetic robots for biomedical applications. Mater. Today Bio.

[54] Yigit B., Alapan Y., Sitti M. (2020). Cohesive self-organization of mobile microrobotic swarms. Soft Matter.

[55] Sharma P.K., Chen C.-Y. (2025). AI-Integrated Micro/Nanorobots for Biomedical Applications: Recent Advances in Design, Fabrication, and Functions. Biosensors.

[56] Mani K., Chen C.-Y. (2021). A smart microfluidic-based fish farm for zebrafish screening. Microfluid. Nanofluid..

[57] Loganathan D., Hsieh C.-L., Ou C.-Y., Chen C.-Y. (2024). A Stepwise Control of Multiple Magnetic Millirobots for Flow Manipulation Applications. Adv. Intell. Syst..

[58] Bray E., Groß R. (2023). Recent Developments in Self-Assembling Multi-Robot Systems. Curr. Robot. Rep..

[59] Sayed M.E., Roberts J.O., Donaldson K., Mahon S.T., Iqbal F., Li B., Franco Aixela S., Mastorakis G., Jonasson E.T., Nemitz M.P. (2022). Modular Robots for Enabling Operations in Unstructured Extreme Environments. Adv. Intell. Syst..

[60] Li X., Xu R., Xie C., Ge Z., Gao B., Lim C.T. (2026). Microscale Architectures for Intelligent Soft Robotics: From Functional Microneedles to Biointegrated Wearable Systems. Nanomicro Lett..

[61] Chen C.-Y., Lin C.-Y., Hu Y.-T., Cheng L.-Y., Hsu C.-C. (2015). Efficient micromixing through artificial cilia actuation with fish-schooling configuration. Chem. Eng. J..

[62] Loganathan D., Ou C.-Y., Hsu C.-W., Chen C.-Y. (2024). A Control Strategy of Multiple Microrobots Using a Hybrid Electromagnetic System. Adv. Mater. Technol..

[63] Loganathan D., Hsieh C.-L., Shi B.-E., Lu Y.-H., Chen C.-Y. (2023). An On-Demand Microrobot with Building Block Design for Flow Manipulation. Adv. Mater. Technol..

[64] Sharma P.K., Wei P.-W., Loganathan D., Lu Y.-H., Chen C.-Y. (2025). Microflow Switching using Artificial Cilia for On-Demand Particle Manipulation. Adv. Intell. Syst..

[65] Loganathan D., OuYang T., Chen C.-Y., Chen C.-Y. (2025). Magnetic Cilia with Programmable Beating Patterns for Vortex-Driven Mixing in Microfluidics. Langmuir.

[66] Sharma P.K., Lu T.-Y., Chen C.-Y. (2025). Microfluidic Mixing with the Dynamic Control of Magnetic Actuation. Adv. Intell. Syst..

[67] Zhong S., Hou Y., Zheng Z., Huang H.W., Shi Q., Huang Q., Fukuda T., Wang H. (2026). Adaptive Shared Cascade Navigation Control of Magnetic Microrobots in Unstructured Dynamic Environments. IEEE Trans. Cybern..

[68] Ongaro F., Pane S., Scheggi S., Misra S. (2019). Design of an Electromagnetic Setup for Independent Three-Dimensional Control of Pairs of Identical and Nonidentical Microrobots. IEEE Trans. Robot..

[69] Lee H.S., Ko Y., Kim C.S. (2025). Enhanced Motion Control of Magnetically Actuated Capsule Robot Using MEMA—A Mobile Electromagnetic Actuation System. IEEE/ASME Trans. Mechatron..

[70] Riad M., Salama I.M. (2020). Electromagnetic Fields and Waves: Fundamentals of Engineering.

[71] Xie H., Sun M., Fan X., Lin Z., Chen W., Wang L., Dong L., He Q. (2019). Reconfigurable magnetic microrobot swarm: Multimode transformation, locomotion, and manipulation. Sci. Robot..

[72] Wang Q., Yang L., Zhang L. (2022). Micromanipulation Using Reconfigurable Self-Assembled Magnetic Droplets with Needle Guidance. IEEE Trans. Autom. Sci. Eng..

[73] Jiang J., Yang L., Hao B., Xu T., Wu X., Zhang L. (2024). Automated Microrobotic Manipulation Using Reconfigurable Magnetic Microswarms. IEEE Trans. Robot..

[74] Xu Z., Ge W., Xu Q. (2025). Reconfigurable robust microrobot collectives with large force output enabled by gradient magnetic fields. Sci. Adv..

[75] Gardi G., Ceron S., Wang W., Petersen K., Sitti M. (2022). Microrobot collectives with reconfigurable morphologies, behaviors, and functions. Nat. Commun..

