# Supplementary material for: Reconfigurable Cilia-Based Magnetic Millirobots for Cooperative Particle Manipulation Through Programmable Assembly in Microfluidics
Source: Micromachines (Basel). 2026 Jul 13;17(7):834. doi: 10.3390/mi17070834 (PMC13413462; doi:10.3390/mi17070834)
Supplement: Supplementary file 1 [file micromachines-17-00834-s001.zip › micromachines-4390181-supplementary.pdf]

Supporting Information

Reconfigurable Cilia-Based Magnetic Millirobots for Cooperative Particle Manipulation Through Programmable Assembly in Microfluidics

*Dineshkumar Loganathan and Chia-Yuan Chen**

This supporting information file includes:

Supplementary Text

S1. Dynamic Response Characterization of the Proposed CMMR Platform

S2. Theoretical Analysis of Magnetic Force and Torque Governing the Self-Organization of CMMRs

S3. Numerical Prediction and Experimental Validation of the Magnetic Field

S4. Particle-Tracking Analysis of Cooperative Particle Transportation

Supplementary Figures

Figure S1. Dynamic response characterization of the proposed CMMR platform under different supplied currents

Figure S2. Numerical prediction and experimental validation of the magnetic field generated by the electromagnetic coil employed for cooperative self-organization of the CMMRs.

Figure S3. Cooperative transportation of a 1600 μm square PDMS particle employing the assembled CMMRs.

Figure S4. Particle-tracking analysis of cooperative particle transportation performed using the assembled CMMRs.

Figure S5. Sequential optical images illustrating the attempted cooperative manipulation of a 2000 μm circular PDMS particle using the assembled CMMRs.

Supplementary Table

Table S1. Comparison of representative magnetic collective robotic systems reported in the literature with the proposed CMMR platform based on reconfigurability, transport velocity, manipulated object characteristics, manipulation mode, manipulation capacity, and cooperative cargo enclosure capability.

Supplementary Text

S1. Dynamic Response Characterization of the Proposed CMMR Platform

To characterize the dynamic response of the proposed CMMR platform, additional experiments were performed by varying the supplied current from 0.1 A to 2.0 A, while quantifying the translational velocity of the CMMRs together with the response delay between electromagnetic coil (EC) activation and the onset of robot motion. The corresponding experimental results are presented in Supplementary Figure S1. As the supplied current increased, the average translational velocity increased from 1.76 ± 0.31 mm s^{-1} at 0.1 A to 2.63 ± 0.32 mm s^{-1}, 2.94 ± 0.34 mm s^{-1}, 3.07 ± 0.28 mm s^{-1}, and 3.50 ± 0.39 mm s^{-1} at 0.5 A, 1.0 A, 1.5 A, and 2.0 A, respectively. Meanwhile, the corresponding response delay decreased from 1.32 ± 0.33 s to 1.06 ± 0.13 s, 0.73 ± 0.34 s, 0.63 ± 0.40 s, and 0.37 ± 0.43 s, respectively. Meanwhile, the assembly and cooperative particle transportation experiments presented in this study were performed using a supplied current of 2 A with a 1 s PWM switching interval between successive EC activations. Under these operating conditions, the measured response delay remained shorter than the switching interval, thereby allowing the CMMRs to respond to each activated EC before the subsequent switching event.

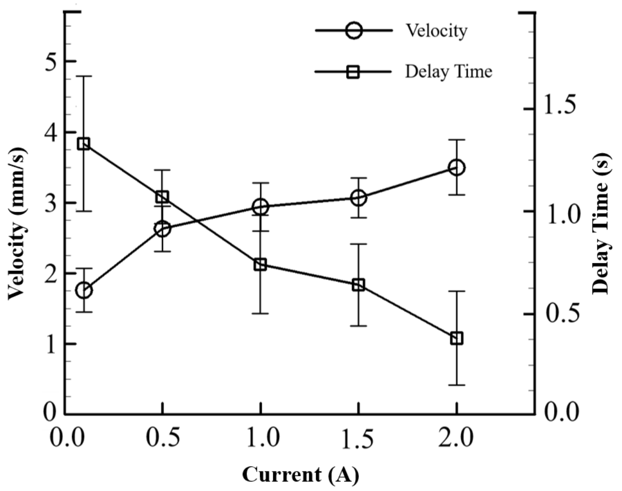

Figure S1. Dynamic response characterization of the proposed CMMR platform under different supplied currents. The average translational velocity and response delay between electromagnetic coil activation and the onset of CMMR motion were experimentally measured by varying the supplied current from 0.1 A to 2.0 A. The data are presented as mean ± standard deviation obtained from five independent experiments.

S2. Theoretical Analysis of Magnetic Force and Torque Governing the Self-Organization of CMMRs

The self-organization of the proposed CMMRs during cooperative assembly was governed by the magnetic force and magnetic torque generated when the permanent magnetic moments embedded within the magnetic cilia were subjected to the externally applied magnetic field produced by the electromagnetic coil array. During the assembly process, the generated magnetic force translated the CMMRs toward the activated electromagnetic coil, whereas the magnetic torque simultaneously reoriented the CMMRs to establish the enclosed reconfigured structure presented in Figure 3A.

The magnetic moment[1] of each magnetic cilium was expressed as

$$m_i = M_i V_i n_i \tag{S1}$$

where M_i is the magnetization of the ith magnetic cilium, V_i is its magnetic volume, and n_i is the corresponding unit magnetization vector.

Since each CMMR consisted of five magnetic cilia, the total magnetic moment of the CMMR was determined by

$$m_{CMMR} = \sum_{i=1}^{5} m_i = \sum_{i=1}^{5} M_i V_i n_i \tag{S2}$$

Upon subjected to the externally generated magnetic field (B), each magnetic cilium will experience a magnetic force[1] given by

$$F_{m,i} = \nabla(m_i \cdot B) \tag{S3}$$

which, for permanent (after magnetizing the cilia) magnetic dipoles, can be expressed as

$$F_{m,i} = (m_i \cdot \nabla)B \tag{S4}$$

Subsequently, the resultant magnetic force acting on the entire CMMR can now be expressed as

$$F_{CMMR} = \sum_{i=1}^{5} F_{m,i} = \sum_{i=1}^{5} (m_i \cdot \nabla)B \tag{S5}$$

This resultant magnetic force was responsible for the translational motion of CMMRs toward the energized electromagnetic coil during the assembly process.

Meanwhile, the magnetic torque[1] acting on each magnetic cilium was expressed as

$$\tau_{m,i} = m_i \times B \tag{S6}$$

whose magnitude was given by

$$\tau_{m,i} = m_i B \sin \theta \tag{S7}$$

where θ denotes the angle between the magnetic moment and the externally applied magnetic field.

Consequently, the total magnetic torque acting on the CMMR can be expressed as

$$\tau_{CMMR} = \sum_{i}^{5} [m_i \times B + (r_i - r_c) \times F_{m,i}] \tag{S8}$$

$$\tau_{CMMR} = \sum_{i=1}^{5} [m_i B \sin \theta + (r_i - r_c) \times F_{m,i}] \tag{S9}$$

where r_i represents the position vector of the ith magnetic cilium and r_c denotes the centroid of the CMMR.

For convenience, the total torque can be expressed as

$$\tau_{CMMR} = \tau_{align} + \tau_{off} \tag{S10}$$

Where τ_{align} represents the alignment torque generated through the interaction between the magnetic moments and the external magnetic field, while τ_{off} corresponds to the torque generated by the off-center magnetic forces acting on the individual magnetic cilia. The combined action of the resultant magnetic force and magnetic torque governed the experimentally observed self-organization process. During assembly, the generated magnetic force translated CMMR 1 and CMMR 3 toward the activated electromagnetic coil, whereas the generated magnetic torque simultaneously rotated both CMMRs until the enclosed reconfigured structure shown in Figure 3A was established. It should be noted that the final assembly orientation was governed by the initial spatial arrangement of the three CMMRs adopted in the present study. Consequently, different initial robot configurations would require different rotational orientations to establish the corresponding enclosed reconfigured structure.

S3. Numerical Prediction and Experimental Validation of the Magnetic Field

To verify the magnetic field responsible for the self-organization of the proposed CMMRs, numerical magnetic field simulations were performed using COMSOL Multiphysics (COMSOL Inc., Burlington, MA, USA). The electromagnetic coil geometry and excitation conditions employed in the numerical model were identical to those utilized in the experimental platform. The corresponding numerically predicted magnetic field distribution generated by a single energized electromagnetic coil is presented in Figure S2B. As illustrated in Figure S2B, the generated magnetic field remained highly localized around the energized electromagnetic coil, where a maximum magnetic flux density of 1.3 mT was predicted at the coil center. The magnetic field gradually decreased with increasing radial distance from the energized coil, thereby generating the magnetic field gradient required to produce translational motion of the CMMRs while simultaneously providing the magnetic torque required for rotational reorientation during cooperative assembly. In adiition, to experimentally validate the numerical prediction, magnetic flux density measurements were performed by employing a Hall-effect sensor positioned at different distances above the energized electromagnetic coil, as illustrated in the inset of Figure S3. The

experimentally measured magnetic flux density exhibited good agreement with the numerical prediction throughout the investigated measurement range. At a separation distance of 1 mm, the experimentally measured magnetic flux density was determined to be 1.25 ± 0.08 mT, whereas the corresponding numerical prediction was 1.30 mT, resulting in a percentage error below 5%. The error bars shown in Figure S3 represent the standard deviation obtained from five independent measurements. Collectively, the theoretical derivation, numerical prediction, and experimental validation provide the physical basis for the translational and rotational behaviors of the CMMRs observed during the cooperative self-organization process.

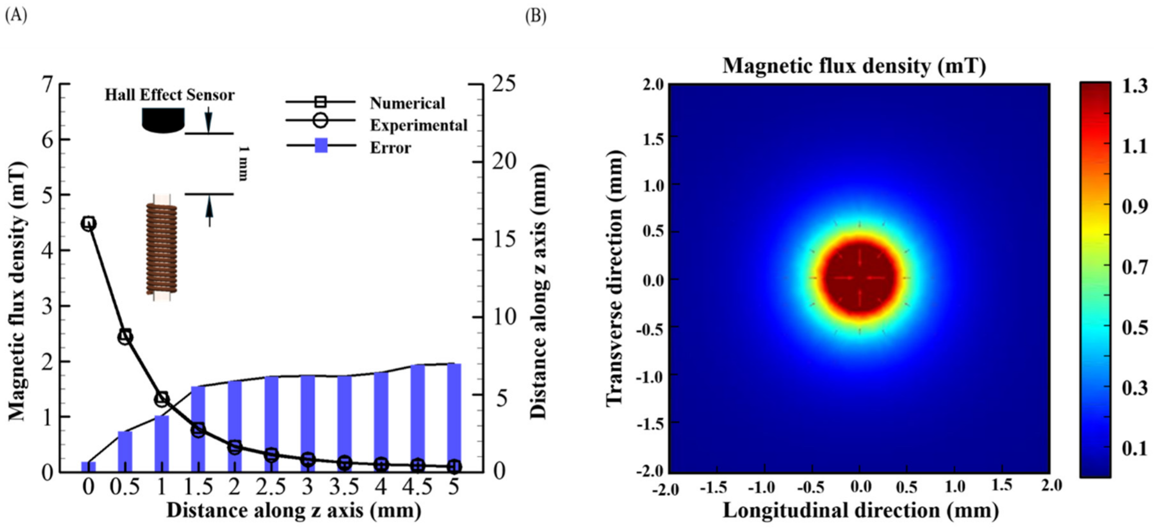

Figure S2. Numerical prediction and experimental validation of the magnetic field generated by the electromagnetic coil employed for cooperative self-organization of the CMMRs. (A) Experimental validation of the numerically predicted magnetic field distribution by employing a Hall-effect sensor (AH3503, Nanjing AH Electronics Co., Ltd., China). The experimentally measured magnetic flux density values exhibited good agreement with the numerical predictions at different distances along the z-axis. At a separation distance of 1 mm, the experimentally measured magnetic flux density was determined to be 1.25 ± 0.08 mT, corresponding to an error of less than 5% relative to the numerically predicted value. The inset illustrates the experimental configuration employed for magnetic field measurement. Error bars represent the standard deviation obtained from five independent measurements. (B) Numerically predicted magnetic flux density distribution generated by a single energized electromagnetic coil positioned beneath the microfluidic platform at a separation distance of 1 mm. A maximum magnetic flux density of 1.30 mT was predicted at the coil center, and the magnetic field gradually decreased with increasing radial distance from the energized coil. The localized magnetic

field distribution established the magnetic field gradient required for translational motion of the CMMRs while simultaneously providing the magnetic torque required for rotational reorientation during cooperative self-organization.

Supplementary Figure S3

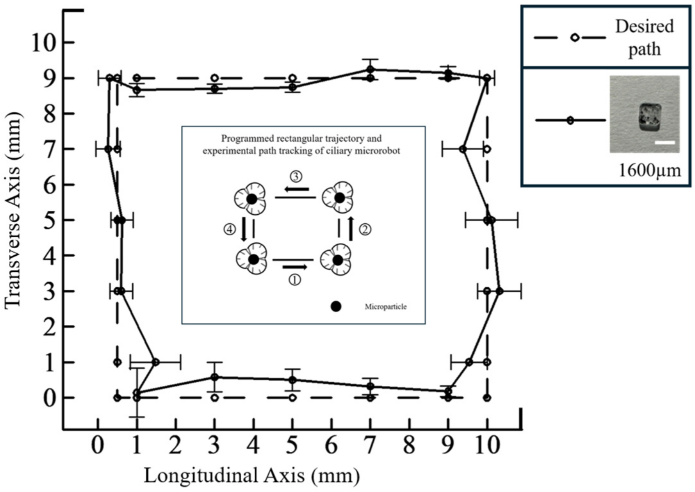

Figure S3. Cooperative transportation of a 1600 μm square PDMS particle employing the assembled CMMRs. The experimentally measured particle trajectory is compared with the prescribed rectangular transportation path, where the particle was sequentially transported along Path 1–Path 4 by the cooperative enclosure formed by the assembled CMMRs. The inset presents the optical image of the employed 1600 μm square PDMS particle. The transportation experiments were repeated five times. The average trajectory deviations were 0.34 ± 0.36 mm, 9.82 ± 0.40 mm, 9.21 ± 0.15 mm, and 0.59 ± 0.36 mm for Path 1–Path 4, respectively, demonstrating the repeatable cooperative transportation of square particles using the proposed CMMR platform.

S4. Particle-Tracking Analysis of Cooperative Particle Transportation

To directly correlate the μPIV flow-field analysis presented in Figure 4B (from manuscript) with the experimentally observed particle transportation behavior, additional particle-tracking experiments were

performed by monitoring the trajectory of the particle confined within the assembled CMMRs during cooperative transportation. The particle was transported along the same pathway investigated in the μPIV measurements, extending from Point A to Point B. The experiment was repeated five times, and the corresponding particle trajectories are presented in **Supplementary Figure S4.** As shown in Supplementary Figure S4, the experimentally measured particle trajectories remained closely confined within the desired transportation corridor throughout the entire transportation process. The average trajectory deviation from the desired transportation path was determined to be 0.23 ± 0.07 mm, demonstrating consistent particle confinement during transportation. These observations directly support the μPIV results presented in Figure 4B, where the assembled CMMRs generated a localized and directional flow field that continuously guided the particle within the central transportation corridor while minimizing lateral displacement. Thus, the particle-tracking analysis provides direct experimental evidence supporting the hydrodynamic mechanism responsible for cooperative particle transportation.

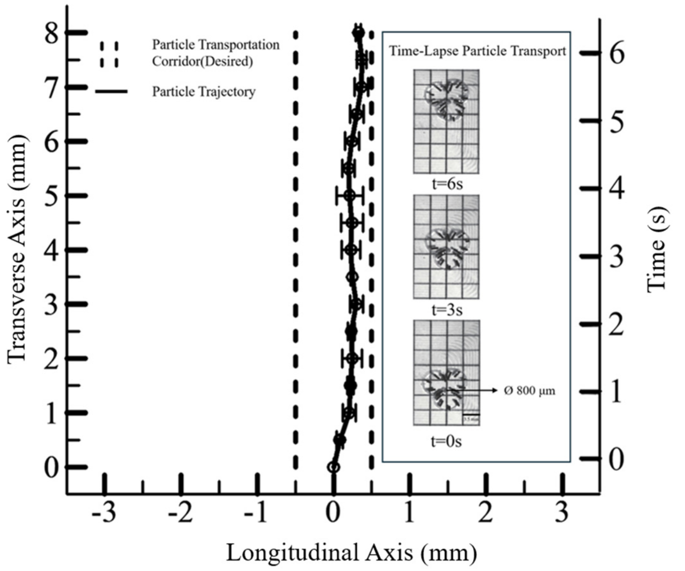

Figure S4. Particle-tracking analysis of cooperative particle transportation performed using the assembled CMMRs. The desired transportation corridor (dashed lines) is compared with the experimentally measured particle trajectories obtained from five independent transportation experiments. The particle was transported from Point A to Point B while confined within the cooperative enclosure formed by the assembled CMMRs. The inset presents representative optical images of the assembled CMMRs transporting the confined particle at t = 0 s, 3 s, and 6 s, illustrating the cooperative transportation process. The scale bar represents 3.5 mm. The measured particle trajectories remained closely confined within the desired transportation corridor, providing direct experimental validation of

the μPIV flow-field analysis presented in Figure 4B.

Supplementary Figure S5

**CMMRs particle grasping process (2000 μm circular-shaped)**	**Time t= 0 s**	**t= 2 s**	**t= 4 s**	**t= 6 s**	**t= 8 s**
	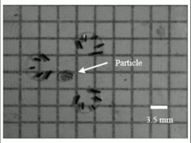	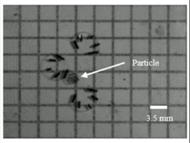	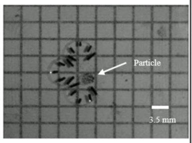	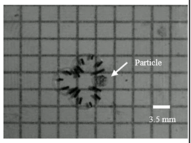	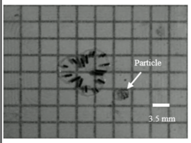

Figure S5. Sequential optical images illustrating the attempted cooperative manipulation of a 2000 μm circular PDMS particle using the assembled CMMRs. The time-lapse images correspond to t = 0, 2, 4, 6, and 8 s during the cooperative capture attempt. The assembled CMMRs approached the particle from different directions and attempted to establish an enclosed cooperative structure for particle capture. However, owing to the large particle size relative to the available cooperative enclosure formed by the three assembled CMMRs, complete enclosure could not be achieved during the initial capture stage. Consequently, the particle escaped from the reconfigured cooperative structure at approximately t = 6 s, preventing successful cooperative manipulation. These observations demonstrate the practical particle manipulation limit of the present three-CMMR cooperative configuration under the investigated operating conditions. The scale bar represents 3.5 mm.

Supplementary Table S1

Table S1. Comparison of representative magnetic collective robotic systems reported in the literature with the proposed CMMR platform based on reconfigurability, transport velocity, manipulated object characteristics, manipulation mode, manipulation capacity, and cooperative cargo enclosure capability.

S.No and [Ref]	System	Reconfigurability	Transport velocity	Manipulated object and size	Manipulation mode	Manipulation capacity	Whether reconfigured structure enclosed the cargo
1 and [2]	Hematite colloidal swarm	Chain, ribbon, and vortex configurations	~30 μm/s	Polystyrene microsphere of size 40μm, Silver microsphere of size 8μm	Collective pushing	One particle per cycle	No
2 and [3]	Magnetic droplets	Reversible shrinkage and expansion	~0.37 mm/s (estimated)	Polypropylene cubic cargo (2*2*1.5 mm^3)	Hydrodynamic trapping	One particle per cycle	No
3 and [4]	Magnetic micro swarms	Vortex and ribbon configurations	~76 μm/s	Polystyrene microbeads of size (75-300μm)	Hydrodynamic trapping	Three particles per cycle	No
4 and [5]	Microrobot collectives	Cilia-like and flat morphologies	Not reported	Droplets, air bubble, 2-mm ABS plastic ball	Collective pushing and hydrodynamic manipulation	5-gram load support	No
5 and [6]	Magnetic micro-disk collectives	X-chain, Y-chain, Gas-like self - propelling pairs	~1.6 mm/s	Polystyrene bed of 1 mm diameter rings, C-shaped, rod-shaped and star-shaped objects	Contact pushing and hydrodynamics manipulation	One particle per cycle	Partially
6. This Work	Cooperative Cilia-Based Magnetic	Three independently controlled	~3.5 mm/s	Circular PDMS particles (500, 800, and 1600 μm) and	Programmable cooperative enclosure,	Single or multiple particles per	Yes (Fully)

	Millirobots (CMMRs)	CMMRs to form an enclosure		square PDMS particle (1600 μm)	transportation, and controlled release	cycle, depending on particle size and available enclosure space	

References:

1. M. Riad, I. M. Salama, in Electromagnetic Fields and Waves: Fundamentals of Engineering, McGraw-Hill Education, **2020**.

2. Xie, H.; Sun, M.; Fan, X.; Lin, Z.; Chen, W.; Wang, L.; Dong, L.; He, Q. Reconfigurable magnetic microrobot swarm: Multimode transformation, locomotion, and manipulation. *Science Robotics* **2019**, *4*, eaav8006.
3. Wang, Q.; Yang, L.; Zhang, L. Micromanipulation Using Reconfigurable Self-Assembled Magnetic Droplets With Needle Guidance. *IEEE Transactions on Automation Science and Engineering* **2022**, *19*, 759–771.
4. Jiang, J.; Yang, L.; Hao, B.; Xu, T.; Wu, X.; Zhang, L. Automated Microrobotic Manipulation Using Reconfigurable Magnetic Microswarms. *IEEE Transactions on Robotics* **2024**, *40*, 3676–3694.
5. Xu, Z.; Ge, W.; Xu, Q. Reconfigurable robust microrobot collectives with large force output enabled by gradient magnetic fields. *Science Advances* **2025**, *11*, eadv9290.
6. Gardi, G.; Ceron, S.; Wang, W.; Petersen, K.; Sitti, M. Microrobot collectives with reconfigurable morphologies, behaviors, and functions. *Nature Communications* **2022**, *13*, 2239.
